# SRS 16‐86 promotes diabetic nephropathy recovery by regulating ferroptosis

**DOI:** 10.1113/EP091520

**Published:** 2024-05-29

**Authors:** Yingchun Qiao, Chao Sun, Shunli Kan, Lu He, Yawen Wang, Huajun Gao, Yingying Zhang, You Cheng, Shuai Wang, Long Zhao, Wenyan Niu

**Affiliations:** ^1^ NHC Key Laboratory of Hormones and Development, Tianjin Key Laboratory of Metabolic Diseases, Department of Clinical Laboratory Chu Hsien‐I Memorial Hospital & Tianjin Institute of Endocrinology, Tianjin Medical University Tianjin China; ^2^ International Science and Technology Cooperation Base of Spinal Cord Injury, Tianjin Key Laboratory of Spine and Spinal Cord Injury, Department of Orthopedics, Tianjin Medical University General Hospital Tianjin Medical University Tianjin China; ^3^ Department of Spine Surgery Tianjin Union Medical Center Tianjin China

**Keywords:** diabetic nephropathy, ferroptosis, ferroptosis inhibitor

## Abstract

Diabetic nephropathy (DN) is a common complication of diabetes mellitus (DM), and cell death plays an important role. Ferroptosis is a recently discovered type of iron‐dependent cell death and one that is different from other kinds of cell death including apoptosis and necrosis. However, ferroptosis has not been described in the context of DN. This study explored the role of ferroptosis in DN pathophysiology and aimed to confirm the efficacy of the ferroptosis inhibitor SRS 16‐86 on DN. Streptozotocin injection was used to establish the DM and DN animal models. To investigate the presence or occurrence of ferroptosis in DN, we assessed the concentrations of iron, reactive oxygen species and specific markers associated with ferroptosis in a rat model of DN. Additionally, we performed haematoxylin–eosin staining, blood biochemistry, urine biochemistry and kidney function analysis to evaluate the efficacy of the ferroptosis inhibitor SRS 16‐86 in ameliorating DN. We found that SRS 16‐86 could improve the recovery of renal function after DN by upregulating glutathione peroxidase 4, glutathione and system x_c_^−^light chain and by downregulating the lipid peroxidation markers and 4‐hydroxynonenal. SRS 16‐86 treatment could improve renal organization after DN. The inflammatory cytokines interleukin 1β and tumour necrosis factor α and intercellular adhesion molecule 1 were significantly decreased following SRS 16‐86 treatment after DN. The results indicate that there is a strong connection between ferroptosis and the pathological mechanism of DN. The efficacy of the ferroptosis inhibitor SRS 16‐86 in DN repair supports its use as a new therapeutic treatment for DN.

## Abstract (highlights)

- **What is the central question of this study?**
  What is the role of ferroptosis in diabetic nephropathy, and what is the efficacy of the ferroptosis inhibitor SRS 16‐86 on diabetic nephropathy.
- **What is the main finding and its importance?**
  SRS 16‐86 could promote the recovery of renal function after diabetic nephropathy by increasing the expression of GPX4, GSH and xCT, and lowering the expression of 4HNE and inflammatory cytokines, which indicates that ferroptosis plays a crucial role in pathophysiological process of diabetic nephropathy, and a ferroptosis inhibitor may be an effective treatment method for diabetic nephropathy.

## 1 INTRODUCTION

Diabetic nephropathy (DN) is a common complication of type 1 and type 2 diabetes mellitus (DM) (Li et al., 2022). DN affects approximately 40% of people with DM and is the primary cause of chronic kidney disease and end‐stage renal disease worldwide (Tu et al., 2021). Glomerular destruction leads to kidney damage, proteinuria and hypertension (Samsu, 2021). The pathogenesis of DN involves several forms of programmed cell death, such as apoptosis, autophagy and necrosis. Specifically, podocyte apoptosis contributes to glomerular injury and podocyte failure, which are closely associated with proteinuria and glomerular structural damage in DN (Barutta et al., 2022). During DN, the epithelial cells of the proximal convoluted tubules also undergo apoptosis, leading to tubular atrophy, the reduction of tubular cells, which ultimately contributes to the loss of renal function (Ma et al., 2022). However, it should be noted that autophagy dysfunction can also lead to the pathogenesis of DN. More specifically, a decrease in podocyte autophagy activity in diabetic kidneys results in alterations in podocyte function, which then destroys the glomerular filtration barrier (Erekat, 2022). Furthermore, the autophagic activity of renal proximal tubular cells in diabetes is weakened, leading to the accumulation of damaged molecules and organelles, which are usually decomposed by autophagy, and this causes proteinuria (Barutta et al., 2023). In addition, necrosis may play a key role in podocyte injury and the subsequent worsening of DN (Morimoto et al., 2020). The pattern of cell death in DN has been intensely researched. Apoptosis and necrosis can cause acute cell injury in DN, and autophagy seems to have beneficial effects on DN. In addition, redox oxidation is involved in DN, and lipid peroxidation, which is correlated with ferroptosis, was reported to be greatly affected. However, the critical role of ferroptosis has not been described in the context of DN.

In 2012, Dixon et al. investigated the mechanism by which erastin induces cell death in cancer cells through renin–angiotensin system (RAS) mutations and formally named this cell death process ferroptosis (Dixon et al., 2012). There was no observable alteration in cell membrane or chromatin morphology during ferroptosis. However, this process is primarily characterized by a reduction in the mitochondrial volume and cristae, and the density of the mitochondrial membrane increased (Basit et al., 2017). Biochemically, the main characteristics of ferroptosis are the decrease of glutathione peroxidase 4 (GPX4) activity, the loss of intracellular glutathione, and the increase of reactive oxygen species (ROS) levels (Zheng et al., 2022). The accumulation of iron, consumption of glutathione and lipid peroxidation are indispensable and occur simultaneously during ferroptosis (Perez et al., 2022). Moreover, there is a histopathological increase in lipid peroxidation and ferroptosis markers in affected women (Ortega et al., 2023). Inhibiting lipid peroxidation can prevent the cell death due to ferroptosis. Ferroptosis is associated with inflammatory processes in which substances released from the cell are significantly involved in the innate immune system and control cellular inflammatory responses, signal transduction and cell proliferation. Ruptured cells release damage‐associated molecular patterns (DAMPs). Ferroptosis leads to the infiltration of macrophages and neutrophils and the release of inflammatory cytokines. When the accumulation of lipid ROS exceeds a certain threshold, a large number of pro‐inflammatory cytokines are produced, such as interleukin 1β (IL‐1β) and tumour necrosis factor α (TNF‐α). Iron overload and lipid ROS have been shown to be the two main factors that cause ferroptosis in tumour cells and brain slices in vitro, which have been reported in DN. Ferroptosis can produce a large amount of ROS, and the kidney is sensitive to oxidative stress due to its rich mitochondrial structure, suggesting that ferroptosis may be related to DN. DN leads to ROS accumulation, and bleeding increases the iron load during this process (Ortega et al., 2023). Glutamic acid could also induce podocyte cell death. After DN, there is an observed increase in the level of glutamic acid, which leads to glutamate excitotoxicity. GPX4 expression is decreased and lipid peroxidation products are increased in the animal model of DN and DN patients’ blood. As previous studies have found that iron chelators can delay the progression of DN, the underlying symptom may be that iron chelators exert protective kidney effects by reducing oxidative stress, inflammation and tubulointerstitial fibrosis. The above studies have confirmed that ferroptosis is related to DN, but the mechanism of ferroptosis leading to DN injury is still unclear. Therefore, we hypothesized that ferroptosis contributes to damage in DN. Inhibiting ferroptosis could reduce functional damage and improve renal repair.

It is worth investigating whether specific inhibitors of ferroptosis can promote renal repair, and in vivo research to identify effective and stable specific inhibitors of ferroptosis is warranted. Ferrostatin 1 (Fer‐1), a first‐generation ferroptosis inhibitor, has been shown to effectively inhibit ferroptosis in vitro. However, its internal functional efficacy is weakened due to its instability in plasma and its metabolism. SRS 16‐86, a third‐generation small‐molecule, has demonstrated the ability to effectively impede lipid ROS production. Notably, it has been observed to exert a potent inhibitory effect on ferroptosis in cases of renal failure resulting from ischaemia–reperfusion injury, and in spinal cord injury, it also shows great potential in inhibiting ferroptosis and reducing inflammation.

Our hypothesis posits that ferroptosis is critical in DN. In particular, our investigation aimed to determine the occurrence of ferroptosis in DN and evaluate the potential of SRS 16‐86 as an inhibitor to enhance renal function recovery. The findings of this study have the potential to improve the understanding of the underlying processes in DN and provide a basis for the development of novel therapeutic approaches.

## 2 METHODS

### 2.1 Animals

Six‐week‐old male Sprague–Dawley rats weighing 160−180 g were purchased from the Laboratory Animal Center of the Academy of Military Medical Sciences (Beijing, China); female rats were not used as they do not respond uniformly to streptozotocin (STZ), which may be related to the oestrogen levels. The animals were placed in a humidity‐ and temperature‐controlled environment, with a light cycle of 12 h, with three animals in each cage with free access to food and water during the 16‐week study period. Experiments were performed under a project licence (No. IRM‐DWLL‐2020192) granted by Ethics Committee of the Institute of Radiation Medicine, Chinese Academy of Medical Science & Peking Union Medical College, in compliance with institutional guidelines for the care and use of animals. The rats were placed into a 10 L euthanasia container and subjected to CO_2_ used at 40% vol/min. The animals’ breathing and eye color were closely observed. CO_2_ was administered for two more minutes after the animals stopped breathing. Then the animals were removed for follow‐up experiments.

### 2.2 Experimental groups

Sprague–Dawley rats were intraperitoneally injected with 60 mg/kg freshly prepared streptozotocin once to cause diabetes, and normal control animals were intraperitoneally injected with the same amount of citric acid buffer. Tail vein blood glucose levels were measured to confirm DM; a random blood glucose level of ≥16.7 mM was used as the standard for the DM model. The dose of SRS 16‐86 was 15 mg/kg/day from week 1 to week 8 after STZ treatment, because this dose of SRS 16‐86 was safe and effective for repairing spinal cord injury in the animal model. We selected six animals from each group at each time point to ensure the accuracy of the data. Animals were randomly assigned to the following groups without exclusions and only the designer of experiment (Y.Q.) was aware of the group allocation at the different stages of the experiment: control group, Sprague–Dawley rats + citrate buffer; DN group, Sprague–Dawley rats + streptozotocin (STZ); and DN‐SRS group, Sprague–Dawley rats + STZ + SRS 16‐86.

All aspects of testing and data analysis were performed in a blinded design.

### 2.3 Iron assay

Renal tissue samples (10 mg) from each group were washed with cold normal saline after being collected. A vibrating homogenizer was immediately used to homogenize the tissue in cold brine. In addition, the collected tissue pieces were homogenized with and ultrasonic cell interfering agent. After 10 min of centrifugation, the supernatant was collected, and the iron concentrations in tissues were determined with an iron content determination kit (BioAssay Systems, Hayward, CA, USA). According to the manufacturer's instructions, a sufficient amount of the working reagents were prepared, the working reagents were transferred to a 96‐well plate, and supernatant was collected from a 96‐well plate. The optical density was determined at a wavelength of 590 nm.

### 2.4 Western blot

Kidneys were collected and placed in a solution (300°C) containing with 20 mM of Tris pH 7.4, 50 mM of NaCl, 1% Triton X‐100, and protease inhibitor 10 μL for homogeneous cracking in cracking liquid. Protein concentrations were measured with the BCA assay. After centrifugation at 13,000 *g* at 4°C for 10 min, the same quantity of protein was extracted with 10% SDS‐PAGE gels and then transferred to a polyvinylidene fluoride membrane for 2 h at 65 V and 4°C with a transfer device. After blocking on a shaking table at room temperature for 2 h, the primary antibody was incubated at 4°C overnight. This was followed by three washes with Tris‐buffered saline with Tween (TBST). After 10 min washing, goat anti‐rabbit immunoglobin G combined with horseradish peroxidase (1:2000; Sigma‐Aldrich, St Louis, MO, USA) was added and incubated for 1.5 h. The membrane was washed with TBST and observed with an enhanced chemiluminescence system. The western blot results were quantified by the ImageJ software (Table 1).

**TABLE 1:** Antibodies used in this study.

Antibody	Company	Dilution
xCT	Abcam	1/2000
GPX4	Abcam	1/2000
4HNE	Thermo Fisher Scientific	1/1000
GAPDH	Abcam	1/1000

Abcam, Waltham, MA, USA; Thermo Fisher Scientific, Waltham, MA, USA. 4HNE, 4‐hydroxynonenal; GAPDH, glyceraldehyde 3‐phosphate dehydrogenase; GPX4, glutathione peroxidase 4; xCT, the light‐chain subunit component of the System; xc^−^, cystine/glutamate antiporter.

### 2.5 ROS and glutathione detection

The kidney homogenate was prepared at a low temperature. A ROS test kit (Beyotime, Shanghai, China) and a total glutathione test kit (Beyotime) were used to determine the ROS level and glutathione (GSH) level, respectively, according to the manufacturer's instructions.

### 2.6 Determination of biochemical indices

Twenty‐four hour urine samples were collected from Sprague–Dawley rats in each group using metabolic cages at 8, 12 and 16 weeks. Urinary biochemistry was detected by a Urinary Microalbumin Test Kit (Ningbo Ruiyuan Biotechnology Co., Ltd, Zhejiang, China), Urinary Creatinine Kit (Ningbo Ruiyuan Biotechnology Co., Ltd) and Urinary Total Protein UC Test Kit (UTP, Ningbo Ruiyuan Biotechnology Co., Ltd). The kidneys and blood samples were extracted after sacrifice. The blood samples were centrifuged at 400 *g* for 10 min to collect the serum. Subsequently we detected creatinine (CRE, Creatinine Test Kit, Baiding, bioengineering (Beijing) Co., Ltd, Beijing, China), uric acid (UA, Uric Acid Test Kit, Beckman Coulter, Brea, CA, USA) and blood urea nitrogen (Urea Nitrogen Test Kit, Beckman Coulter) levels in the serum of Sprague–Dawley rats in each group. Urinary biochemistry was detected by a (Roche, Basel, Switzerland) Biochemical Analyzer and Blood biochemistry was detected by a Beckman Biochemistry Analyzer.

### 2.7 Haematoxylin and eosin staining

Hematoxylin and eosin (HE) staining was used to evaluate the histological structure of each group. After euthanizing with CO_2_ used at 40% vol/min, the rats were perfused with 200 mL 0.9% NaCl and 100 mL 4% paraformaldehyde through their hearts. Kidney tissue was extracted and soaked in 4% paraformaldehyde at 4°C for 1 day. Then, paraffin‐embedded, 5 μm‐thick sections were stained with HE. The sections were visualized using a light microscope at ×200. Injury score was classified based on degree of kidney damage and the following criteria were used for the assessment of injuries. Renal tubule injury: score 0: no damage such as renal tubular atrophy; score 1: no more than 25% cortical renal tubular atrophy or vacuolar changes, and tubular epithelial cell detachment or necrosis; score 2: 26%−50% cortical renal tubular atrophy or vacuolar changes, and tubular epithelial cell detachment or necrosis; score 3: more than 50% cortical renal tubular atrophy or vacuolar changes, and tubular epithelial cell detachment or necrosis. Glomerular injury: score 0: no characteristic glomerular damage. Score 1: Widening of the mesangial matrix, thickening of the glomerular capillary basement membrane with or without stratification. No more than 25% non‐sclerotic glomeruli (at least moderate mesangial increase) are affected; score 2: widening of mesangial matrix and thickening of glomerular capillary basement membrane, 26%–50% non‐sclerotic glomeruli (at least moderate mesangial increase) are affected; score 3: more than 50% of non‐sclerotic glomeruli (at least moderate mesangial increase) exhibit widening of the mesangial matrix and thickening of the glomerular capillary basement membrane. Renal interstitial fibrosis: score 0: interstitial fibrosis accounts for no more than 5% of cortical areas; score 1: mild, cortical areas with interstitial fibrosis account for 6%−25%; score 2: moderate, interstitial fibrosis accounts for 26%−50% of cortical areas; score 3: severe, cortical areas with interstitial fibrosis exceeding 50%. Thirty non‐confluent view fields were collected from each kidney (i.e., from each animal), and the average of the percentages that meet the damage criteria was calculated for analysis.

### 2.8 Statistical analysis

Statistical analysis was conducted using GraphPad Prism 9.0 software (GraphPad Software, Boston, MA, USA). One‐way ANOVA was used for comparison among multiple groups, and the Bonferroni correction was performed. Data are expressed as means ± standard deviation of the SD. The difference was considered statistically significant at a *P*‐value < 0.05.

## 3 RESULTS

### 3.1 Establishment of DM and DN animal models

**FIGURE 1:**
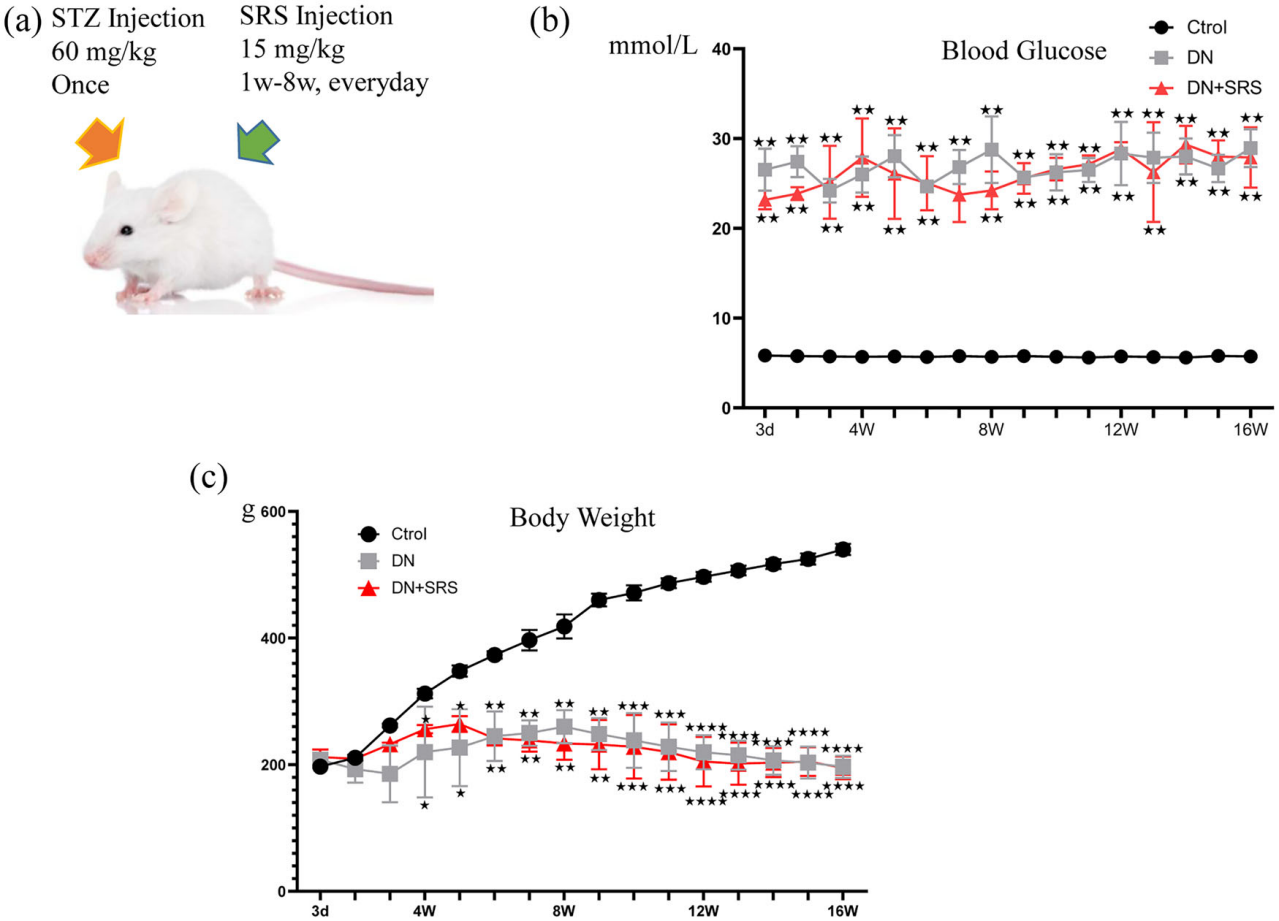
Establishment of the DM and DN animal models. (a) A schematic representation of the animal model. (b, c) Blood glucose (b) and body weight (c) after STZ treatment. Data are shown as means ± SD; *n* = 6/group.

**FIGURE 2:**
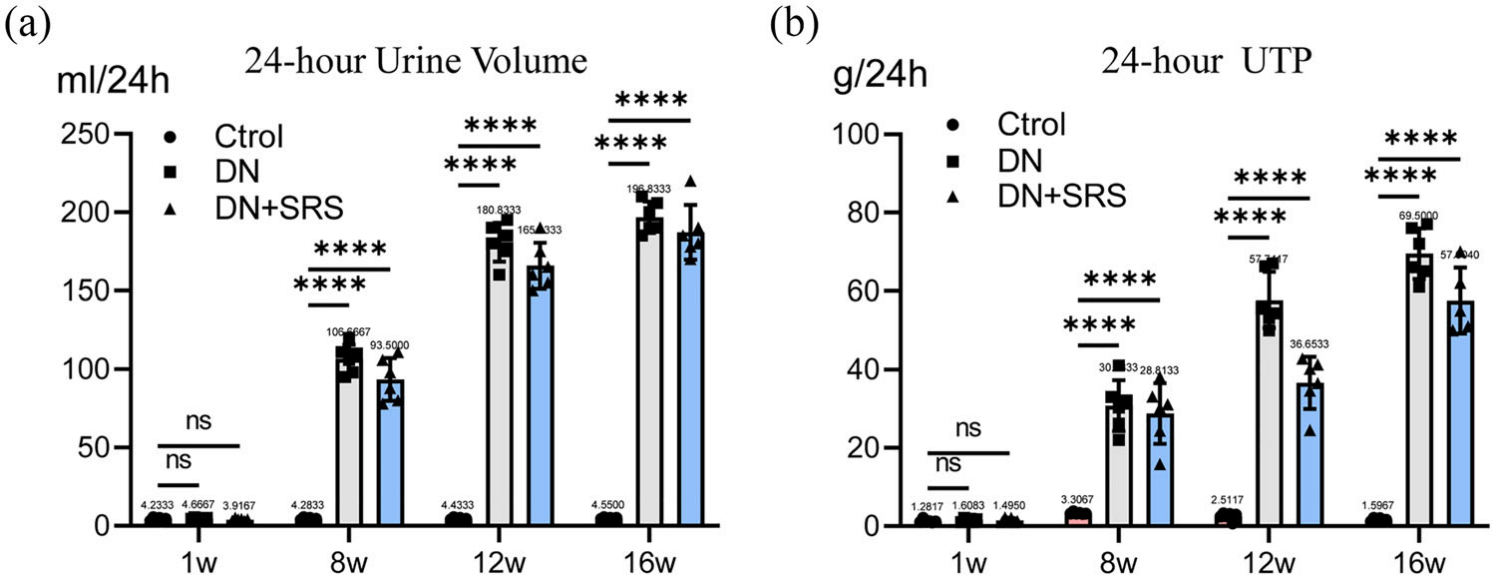
Urinary biochemical indices were used to evaluate renal function. Twenty‐four hour urine volume (a) and 24‐h UTP (b) after STZ treatment. (*n* = 6/group; ns: *P *> 0.05; ****P < 0.0001.)

We induced diabetes models according to the protocols of the Animal Models of Diabetic Complications Consortium. Diabetes was induced in Sprague–Dawley rats weighing 160−180 g with a single intraperitoneal injection of freshly prepared STZ (60 mg/kg) (Figure 1a). The DN‐SRS group was then treated with intraperitoneal injection of 15 mg/kg/day SRS 16‐86 from week 1 to week 8 after STZ treatment, while the DN group was treated with the same dose of dimethyl sulfoxide (Figure 1a). Blood glucose in the DN and DN‐SRS groups was significantly increased compared to that in the control group 3 days after STZ treatment. Additionally, both the DN and DN‐SRS groups exhibited a significant decrease in body weight in comparison to the control group, thereby confirming the successful establishment of the diabetic animal model (Figure 1b, c). We evaluated changes in renal function by measuring urine biochemical indicators including 24‐h urine volume and 24‐h urinary total protein (UTP). As shown in Figure 2, 24‐h urine volume and 24‐h UTP in the DN and DN‐SRS groups were markedly higher than those of control group at the 8‐week time point, indicating that the DN animal model was successfully constructed (Figure 2a, b).

### 3.2 Reduced iron overload by ferroptosis inhibition

**FIGURE 3:**
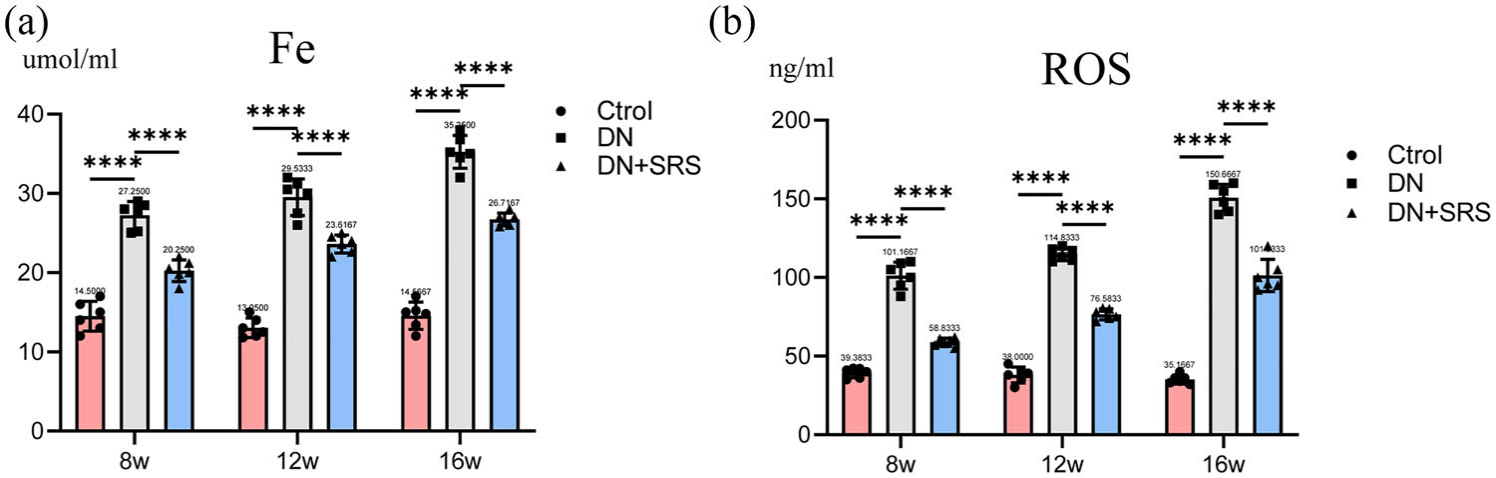
Reduced iron overload and ROS by ferroptosis inhibition. The content of iron overload (a) and expression of ROS (b) in all groups. (*n* = 6/group; ****P < 0.0001.)

Iron overload plays an important role in oxidative stress, and the accumulation and lipid peroxidation of Fe^2+^ are the key factors in ferroptosis. To explore the effect of SRS 16‐86 on iron overload in DN, an iron ion detection kit was used to measure the iron content. In this study, the iron overload in the DN group was significantly increased compared with that in the control group, while SRS 16‐86 treatment reduced iron level compared with those in the DN group, indicating a reduction in iron overload (Figure 3a).

### 3.3 SRS 16‐86 decreased ROS, MDA and 4‐hydroxynonenal level in DN

**FIGURE 4:**
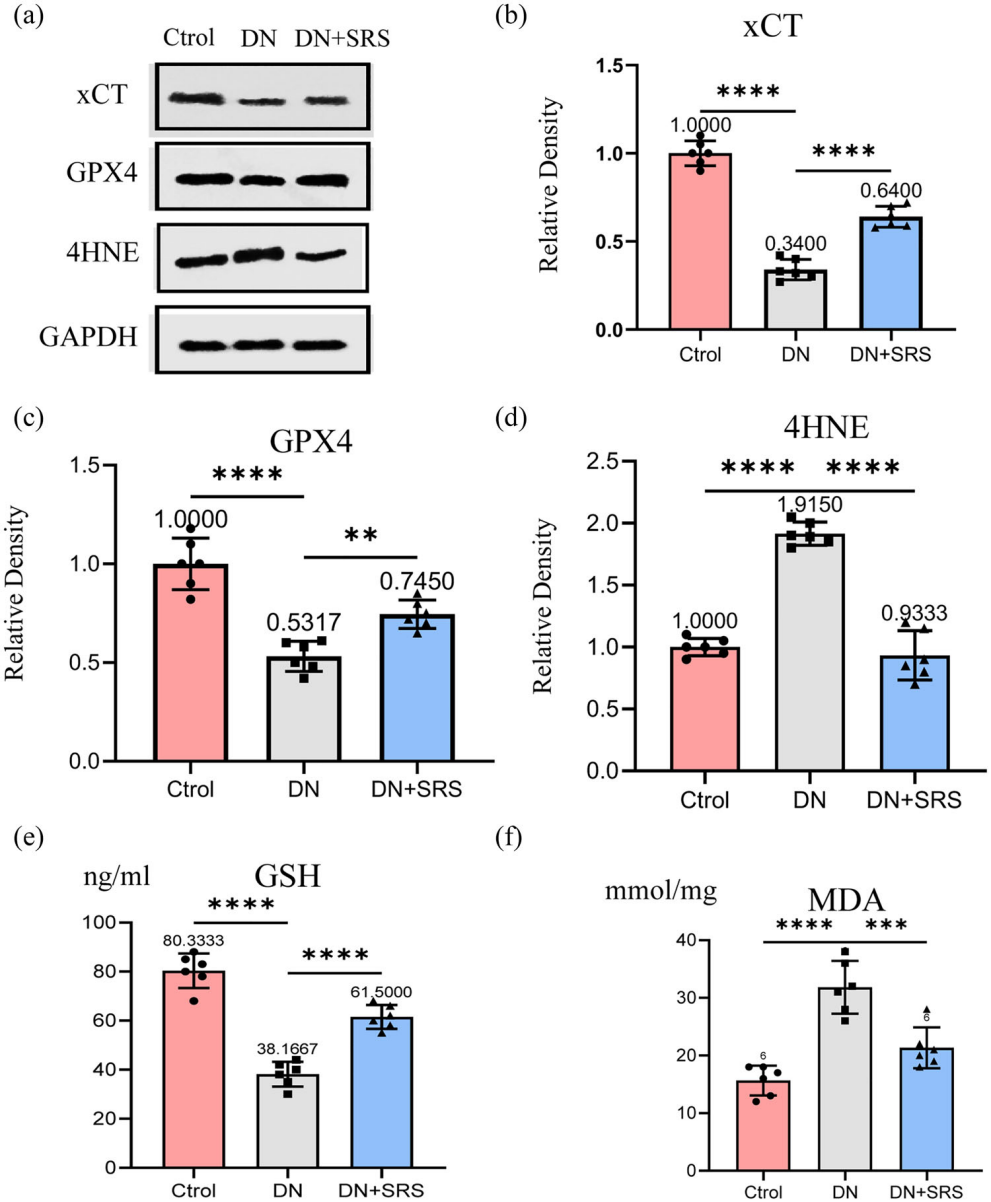
Expression changes of xCT–GSH–GPX4–4HNE pathway. (a) Representative western blot images of xCT, GPX4 and 4‐HNE. (b–d) Quantifications of western blot band of xCT (b), GPX4 (c) and 4‐HNE (d). (e) Quantification of GSH assessment. (f) Expression of MDA. (*n* = 6/group; **P < 0.01; ***P < 0.001; ****P < 0.0001.)

Ferroptosis is caused by the lipid peroxidation induced by ROS accumulation: ROS decomposition produces malondialdehyde (MDA) and 4‐hydroxynonenal (4HNE), which then form covalent adjuncts with proteins, DNA, lipids, and other macromolecules to cross‐link and inactivate proteins that promote ferroptosis, thus precipitating cell membrane rupture and ferroptosis. In this study, we detected ROS (Figure 3b), MDA (Figure 4f) and 4HNE (Figure 4a, d) expression. 4HNE, MDA and ROS levels were significantly upregulated post‐DN in the DN group compared to the control group. After treatment with SRS 16‐86, 4HNE, MDA and ROS expression were decreased, demonstrating that SRS 16‐86 could inhibit lipid peroxidation.

### 3.4 SRS 16‐86 upregulated the levels of xCT, GSH and GPX4 in DN

The xCT–GSH–GPX4 axis contributed to the negative regulation of ferroptoptic‐related cell death. High extracellular glutamate levels can inhibit systemic xCT activity and thus induce ferroptosis. We detected the expression of xCT, GPX4 and GSH post‐DN (Figure 4a–c, e). xCT, GPX4 and GSH levels were significantly reduced post‐DN in the DN group compared to the control group. These results indicated that the capacity of peroxidation repair in the kidneys of diabetic rats was dramatically reduced. We also found that the expression of xCT, GPX4 and GSH was upregulated after treatment with SRS 16‐86. The antioxidant capacity was repaired in the SRS group.

### 3.5 The ferroptosis inhibitor reduced DN inflammation

**FIGURE 5:**
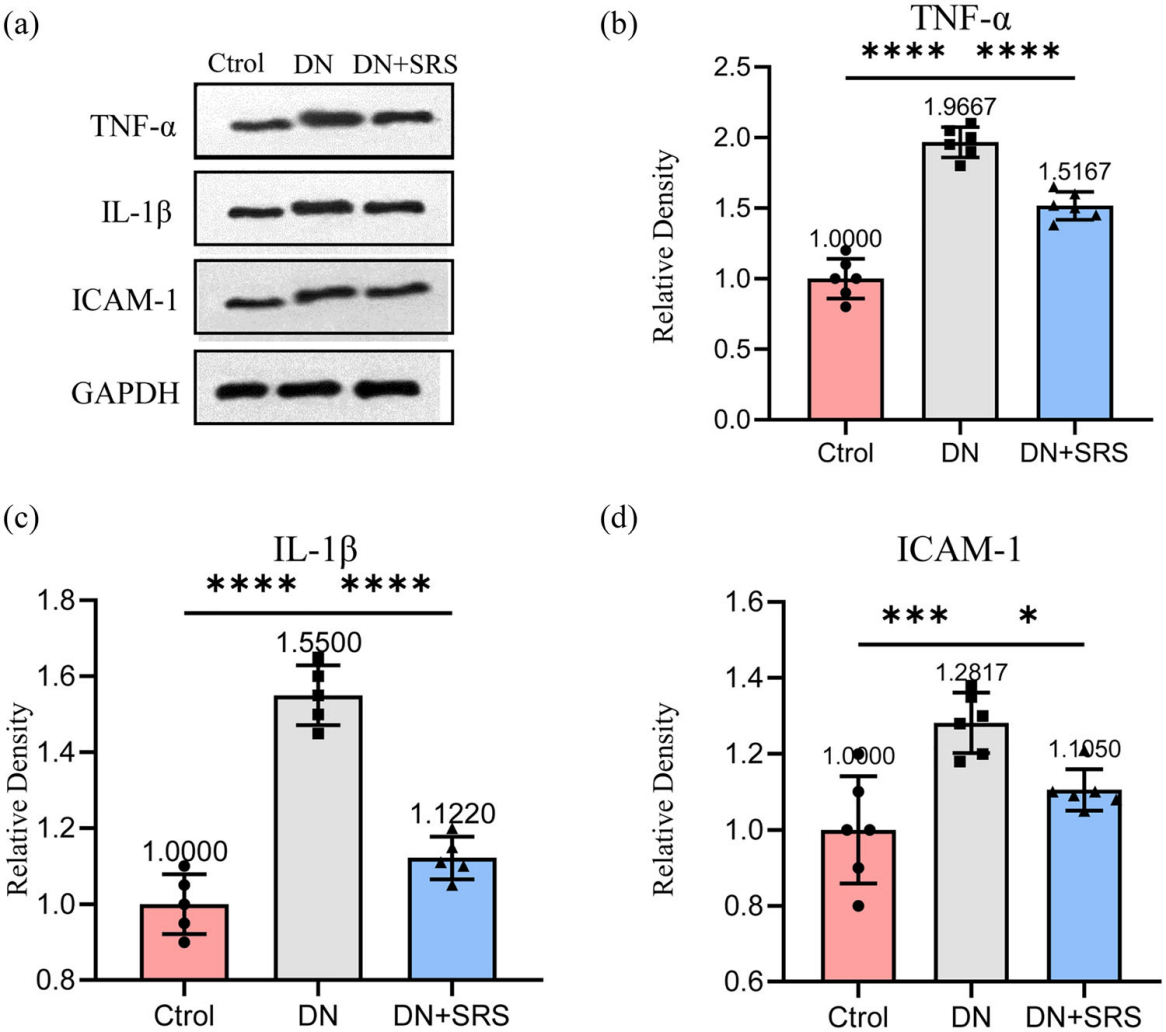
Reduction of DN inflammation by ferroptosis inhibitor. (a) Representative western blot images of TNF‐α, IL‐1β and ICAM‐1. (b–d) Quantification of western blot band of TNF‐α (b), IL‐1β (c) and ICAM‐1 (d). (*n* = 6/group; *P < 0.05; ***P < 0.001; ****P < 0.0001.)

Ferroptosis is involved in mediating the inflammatory response. The expression of IL‐1β, TNF‐α, and intercellular adhesion molecule 1 (ICAM‐1) were assessed by western blotting post‐DN to clarify the amount of inflammation (Figure 5a–d). SRS 16‐86 attenuated the levels of these factors. SRS 16‐86 decreased the levels of inflammatory cytokines in the kidneys of rats with DN.

### 3.6 Ferroptosis inhibition improved organizational structure after DN

**FIGURE 6:**
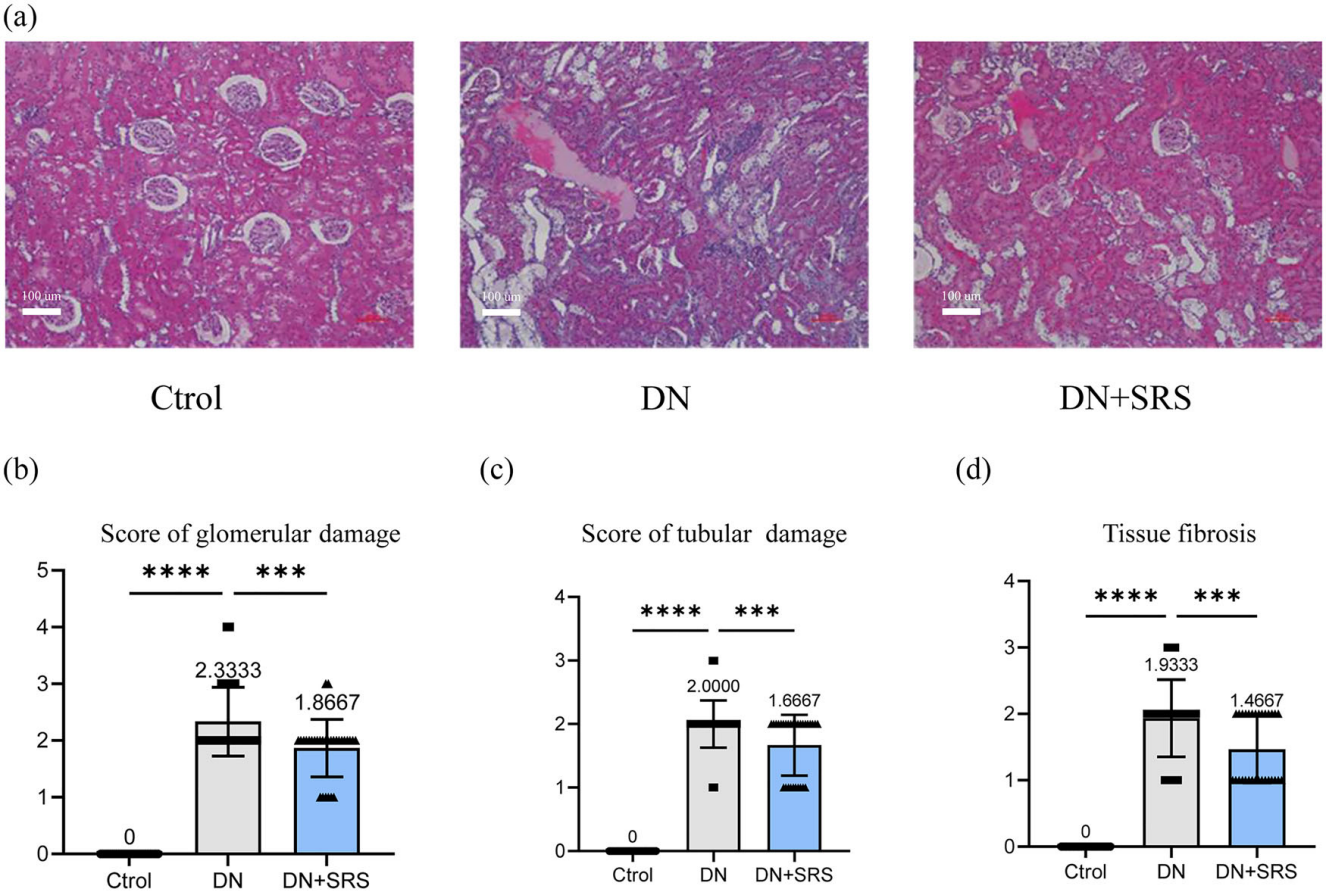
(a) The renal organizational structure in control group, DN group and DN‐SRS group. (b–d) The score of glomerular and tubular damage, as well as fibrosis in control group, DN group and DN‐SRS group. (*n* = 6/group; ***P < 0.001; ****P < 0.0001.)

To explore whether SRS 16‐86 modulates the organizational structure after DN, we examined the kidneys of rats in each group by HE staining (Figure 6). In comparison to kidneys in the control group, obvious disorder was visible in kidney sections in the DN group. In the DN group, the glomerular morphology was abnormal, the renal tubule space was enlarged and renal tissue fibrosis was enhanced. Moreover, the DN‐SRS group showed an improvement of organizational structure when compared with the DN group. These results show that SRS 16‐86 significantly reduced destruction of the organizational structure as evidenced by the increased amount of normal tissue and the decreased extent of lesions, which were associated with improved renal function recovery.

### 3.7 Ferroptosis inhibition improved renal function recovery after DN

**FIGURE 7:**
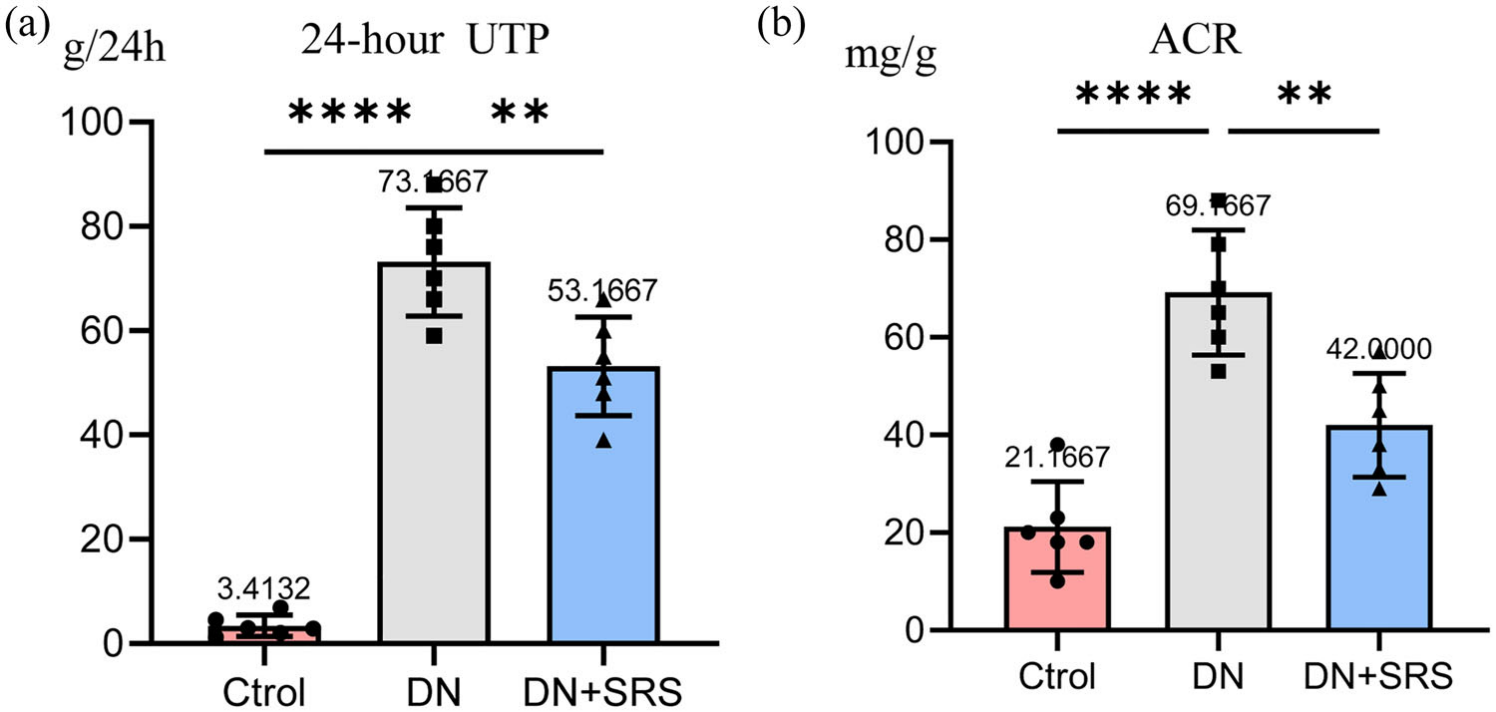
Improved renal function recovery after DN via the inhibition of ferroptosis. Urine biochemical indicators including 24‐h UTP (a) and ACR (b) in the DN‐SRS group were markedly lower than those in the DN group (*n* = 6/group; **P < 0.01; ****P < 0.0001.)

**FIGURE 8:**
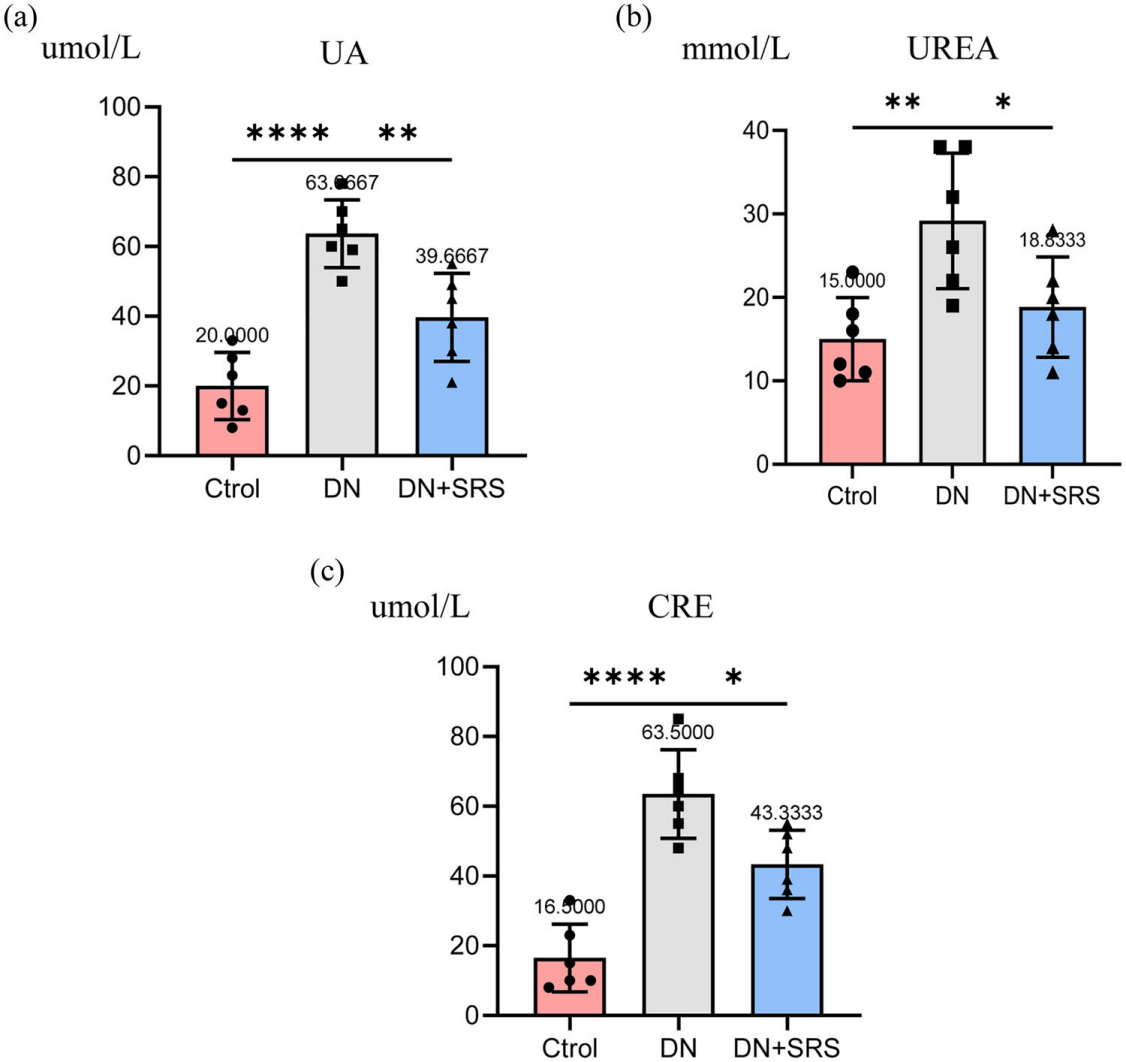
Improved renal function recovery after DN via the inhibition of ferroptosis. The blood biochemical indicators including UA (a), urea (b) and creatinine (CRE) (c) in the DN‐SRS group were markedly lower than that in the DN group. (*n* = 6/group; *P < 0.05; **P < 0.01; ****P < 0.0001.)

To explore whether SRS 16‐86 could improve renal function recovery after DN, we examined 24‐h UTP and Albumin to Creatinine ratio (ACR) content in urine, and uric acid (UA), urea and creatinine levels in blood (Figures 7a,b and 8a–c). As is shown in Figure 7, 24‐h UTP and ACR in the DN‐SRS group were markedly lower than those in the DN group. We observed the same changes in serum UA, urea and creatinine. These results showed SRS 16‐86 significantly promoted renal function recovery after DN.

## 4 DISCUSSION

Ferroptosis is a newly discovered cell death pathway that has been confirmed in stroke, Parkinson's disease and spinal cord injury (Qiao et al., 2019; Zhang et al., 2019). However, the role of ferroptosis was first found in the injury in a renal ischaemic–reperfusion model, and there is currently a lack of research on the key role of ferroptosis in DN. Our study revealed a decrease in crucial regulatory factors associated with ferroptosis, namely, GPX4, GSH and xCT, in the context of DN. Moreover, analysis of ROS and 4HNE indicated an elevation in levels of lipid peroxidation. Subsequent examination of tissue structure and renal function following the administration of the ferroptosis inhibitor SRS 16‐86 revealed a notable enhancement in the preservation of normal tissue structure and the restoration of renal function. Additionally, treatment with SRS 16‐86 led to a reduction in inflammatory cytokines. These findings provide evidence supporting the beneficial effects of inhibiting ferroptosis in DN.

Because the ferroptosis process is accompanied by excessive lipid ROS production, which can lead to oxidative stress, and the mitochondrial‐rich kidney structure is more vulnerable to oxidative stress damage, the traditional pathogenesis of DN is also involved in oxidative stress, which suggests that ferroptosis may be associated with DN. Excess iron in tissue and cells induces cell death by producing ROS through the Fenton reaction. Additionally, GPX4 inactivation due to GSH depletion can also lead to ROS accumulation through lipid peroxidation (Ashraf et al., 2020; Zhang & Li, 2022). ROS can react with polyunsaturated fatty acids in lipid membranes and induce lipid peroxidation. A study has shown that ferroptosis is closely regulated by the combination of several signalling pathways, including the regulation of iron homeostasis, the RAS/rapidly accelerated fibrosarcoma (RAF) signalling pathway and the glutamine–cystine transport signalling pathway (Sugezawa et al., 2022; Zhang et al., 2023). Factors such as GSH and GPX4 are pivotal in ferroptosis. GSH efficiently eliminates excessive ROS from the organism through the action of GPX4, thereby providing protection against potential harm. Nevertheless, the disruption of the delicate balance among GSH, GPX4 and ROS leads to the inability to promptly eliminate the surplus ROS, consequently resulting in adverse consequences. Notably, a deficiency in GPX4 has been demonstrated to significantly enhance iron‐induced cell death in the epithelial cells of the renal tubules, ultimately leading to the development of acute renal failure (Ma et al., 2022). It has been found that an increase of glutamate can promote the activation of the glutamate–glutathione transporter, allowing glutamate to enter the cell to produce excessive ROS and induce ferroptosis (Xu et al., 2022).

Ferroptosis is related to the action of active iron, lipid peroxidation and the weakening of antioxidant capacity and intervention in these processes may achieve the therapeutic purpose of inhibiting ferroptosis. Current evidence suggests that the ACSL4 inhibitor rosiglitazone reduces pathological kidney damage by reducing lipid peroxidation (Xie et al., 2022). And iron chelating agents are also an effective way to inhibit iron death by reducing excess intracellular iron, since the occurrence of iron death depends on excess intracellular iron producing large amounts of ROS through the Fenton reaction. SRS 16‐86 is a newly more stable and effective synthesized iron‐droop inhibitor (Dixon et al., 2014). This compound has a strong protective effect against renal ischaemia–reperfusion injury. In our DN model, SRS 16‐86 increased the concentration of GSH in renal tissue and decreased the lipid ROS marker 4HNE. GPX4 and xCT are markers of ferroptosis. The expression of GPX4 and xCT was downregulated after injury and increased after inhibitor treatment. These results indicate that SRS 16‐86 inhibits the ferroptosis process. HE staining showed that more tissue was retained after inhibitor treatment. Study showed that DN damaged the renal tubules via the hypoxia‐inducible factor (HIF)‐1/haem oxygenase (HO)‐1 pathway (Souza et al., 2020). Increased haem decomposition causes iron to build up in the renal tubules, which in turn leads to a rise in ROS production and an accumulation of lipid peroxidation. On the contrary, however, some studies have demonstrated that (Li et al., 2021; Lin et al., 2023) HO‐1 protects renal epithelial cells from oxidative stress in a significant way. Moreover, previous studies showed that (Haythorne et al., 2019; Lima et al., 2022) due to the special reabsorption function of renal tubular tissue (including glucose and iron), which contains numerous mitochondria, its metabolic activity and energy demand are high; diabetes induces impairments in mitochondrial energy metabolism, which result in significant intrarenal oxidative stress and cell damage. Ferroptosis often occurs in renal tubules during the development of renal diseases because of the sensitivity of renal tubular tissue to oxidative stress and lipid peroxidation. And our study also suggested that ferroptosis is closely related to oxidative stress as well as cell damage. Therefore, renal tubular tissue may be more likely to be affected by ferroptosis in diabetes. More in‐depth research remains to be done.

Inflammation is an immune response to alterations in the internal and external environment of cells. A balanced immune microenvironment can be protective, whereas microenvironment imbalance can result in detrimental effects. The occurrence of ferroptosis is frequently accompanied by inflammation (Fan et al., 2021; Qi et al., 2020; Shi et al., 2022). DN is widely recognized as a chronic inflammatory disease. The process of ferroptosis is accompanied by changes in DAMPs and inflammatory factors. DAMPs lead to the infiltration of renal inflammatory cells through the immune response and release inflammatory factors, amplifying the immune response, leading to a sustained inflammatory response, and are associated with the progression of DN. In a mouse model of folic acid‐induced acute renal injury, necrosis and inflammation accompanied by ferroptosis led to the death of a large number of renal tubular cells, causing acute renal failure and early death (Astudillo et al., 2023). In our study, the administration of SRS 16‐86 treatment resulted in a decrease in the expression of proinflammatory cytokine IL‐1β, TNF‐α and ICAM‐1. This observation suggests that inhibiting ferroptosis may impede the progression of the inflammatory cascade in DN. It is worth noting that lipid peroxidation in ferroptosis can generate inflammatory signalling molecules. These findings align with the previously observed effects of Fer‐1 in reducing proinflammatory cytokines in an acute renal injury model. However, the relationship between ferroptosis and the inflammatory microenvironment of DN necessitates further investigation. Indications that the ferroptosis pathway is related to the injury of DN has opened up exploration of this problem. First, GSH exhaustion and lipid peroxidation have been observed in DN, but GPX4 and other essential factors associated with iron removal in DN are still unclear. The exploring of these factors could provide new ways to understand the pathophysiology of DN. Second, moreover, the sensitivity of the different types of cells in DN to ferroptosis is unclear; it will be interesting to investigate the sensitive cell types when studying ferroptosis in DN in the future. Furthermore, the injection time window and concentration of SRS 16‐86 need to be certificated in following research for clinical use in the future. In our study, we observed abnormal glomerular morphology, enlarged intratubular spaces and increased renal fibrosis in the DN group. However, the precise impact on cellular constituents within each tissue remains unclear. It is important to determine whether other known drugs could promote DN by inhibiting ferroptosis. Studying ferroptosis may elucidate the mechanism of traditional medicine and provide a new direction for treatment.

### 4.1 Conclusions

In conclusion, we have demonstrated that ferroptosis plays an important role in pathophysiological process of DN by using SRS 16‐86 to treat DN. This inhibitor may be an effective agent for treating patients with DN.

## AUTHOR CONTRIBUTIONS

Conception and design: All authors. Administrative support: Wenyan Niu. Provision of study materials or patients: Yingchun Qiao, Chao Sun, Shunli Kan, Lu He, Yawen Wang, Huajun Gao, Yingying Zhang, You Cheng, Shuai Wang, and Long Zhao. Collection and assembly of data: Yingchun Qiao, Chao Sun, Shunli Kan, Lu He, Yawen Wang, Huajun Gao, Yingying Zhang, You Cheng, Shuai Wang, and Long Zhao. Data analysis and interpretation: Yingchun Qiao, Chao Sun, Shunli Kan. Manuscript writing: All authors. All authors have read and approved the final version of this manuscript and agree to be accountable for all aspects of the work in ensuring that questions related to the accuracy or integrity of any part of the work are appropriately investigated and resolved. All persons designated as authors qualify for authorship, and all those who qualify for authorship are listed.

## CONFLICT OF INTEREST

The authors have no conflicts of interest to declare.

## Data Availability

Data available on request from the corresponding author.
